# A Large Gradually Increasing Cutaneous Swelling on Lower Back

**DOI:** 10.4103/0974-2077.63300

**Published:** 2010

**Authors:** GK Sawke, Nilima Sawke, BS Gaur, S Thakur

**Affiliations:** *Department of Pathology, People's College of Medical Sciences and Research Centre, Bhopal, Madhya Pradesh, India*

A 60-year-old female presented with a large, gradually increasing swelling on her lower back, present since the last five years. The swelling was slightly tender, firm and nodular. There was no history of hypertension, diabetes, or tuberculosis. The mass was excised and sent for histopathological examination.

**Histopathology:** The cut section showed grey-white and brown-lobular areas with infiltrating margins in the subcutaneous tissue [Figure 1]. Multiple sections were studied from tumor areas and surgical margins.

**Figure 1:**
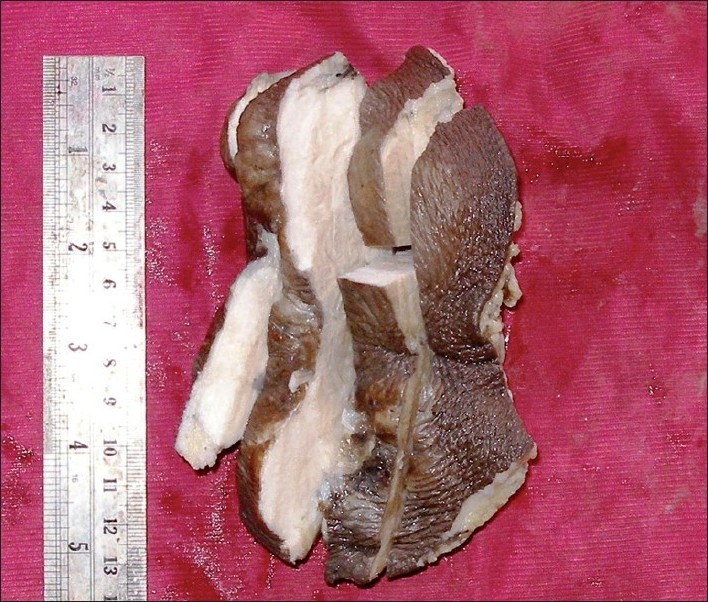
Gross examination reveals a grey white nodular cutaneous mass

The sections studied showed mostly uniform, spindle-shaped cells, arranged in a storiform pattern. The nuclei were oval to elongated and regular, but at places showed pleomorphism, hyperchromasia and irregularly clumped chromatin. Few mitotic figures and occasional bizarre cells were also seen, with minimal inflammatory infiltrates. A deep extension of tumour cells was seen in the subcutaneous fat. There was a thinning of the overlying epidermis [Figures 2 – 4].

**Figure 2:**
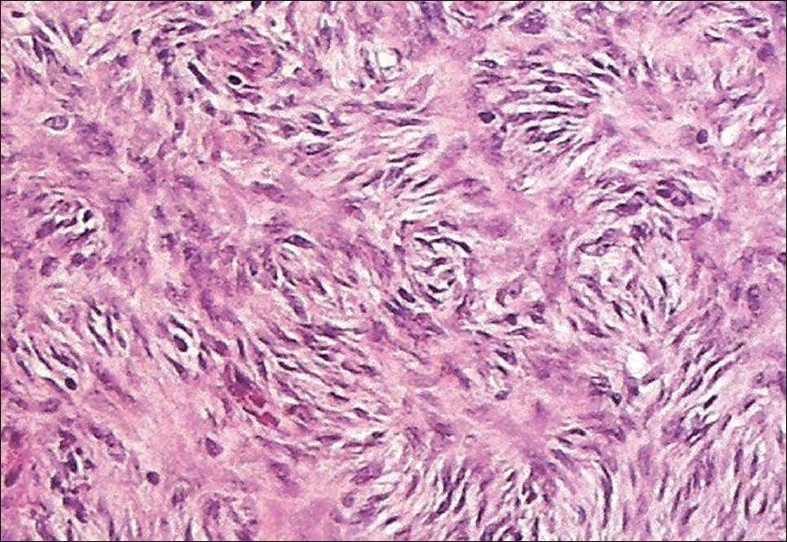
Spindle shaped cells are seen arranged in a storiform pattern [Hematoxylin and eosin, ×40]

**Figure 3:**
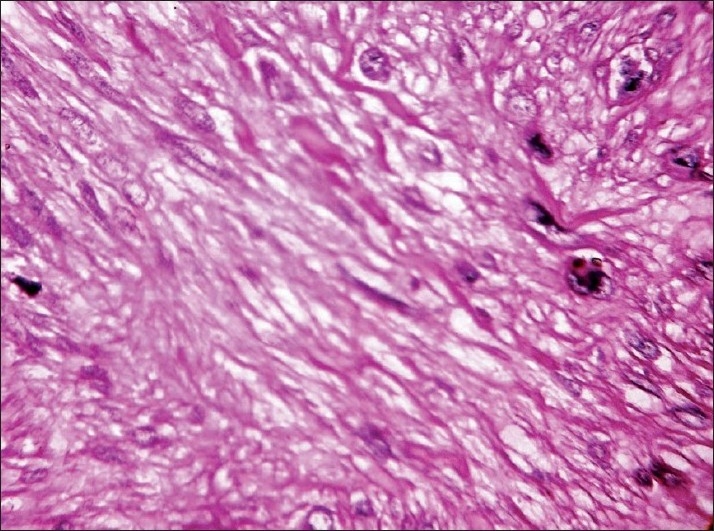
Subcutaneous tissue is seen infiltrated by the spindle shaped cells [Hematoxylin and eosin, ×40]

**Figure 4:**
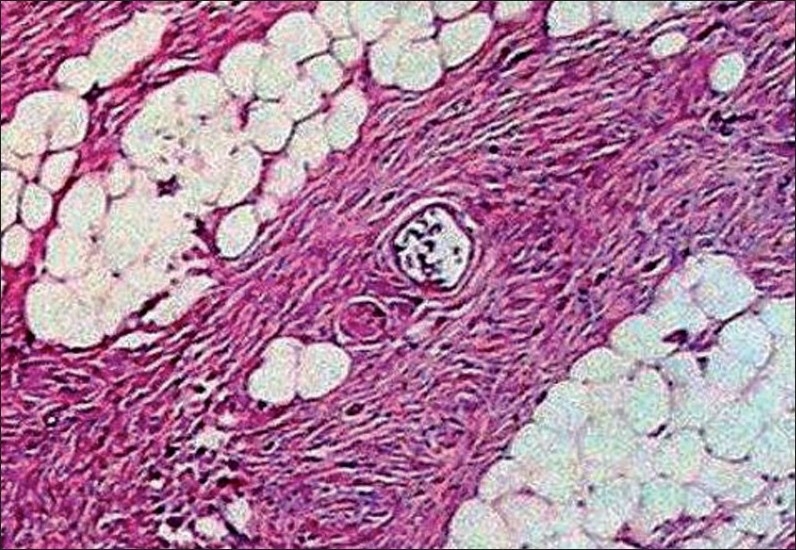
Higher magnification reveals cells with oval to elongated, regular nuclei which at places show pleomorphism, hyperchromasia and irregularly clumped chromatin (Hematoxylin and Eosin, ×40)

## WHAT IS YOUR DIAGNOSIS?

### DIAGNOSIS: DERMATOFIBROSARCOMA PROTUBERANS

#### Discussion

Dermatofibrosarcoma protuberans (DFSP) was first described by Darrier and Ferran in 1924, as a progressive and recurrent dermatofibrosarcoma.[1] The term dermatofibrosarcoma protuberans was first used in 1925 by Hoffman. DFSP is a fibrohistiocytic tumor, of intermediate malignant potential, characterized by a distinctive storiform growth pattern and frequent local recurrences. The tumor accounts for less than 0.01% of all malignancies.[1–3] It is common in early and middle adult life, with a peak between the second and third decades. A majority of patients are under 40 years of age at the time of diagnosis. Males are affected more than females.[1,4] Sex distribution varies among the published series.[5] The most common site of DFSP in decreasing frequency is, trunk 47.4%, lower extremity 19.9%, upper extremity 18.2%, and head and neck region 14.5%.[1] Involvement of the hands and feet is rare.[2,3,5]

The cause of DFSP is not known, although cytogenetic studies have shown tumor cells with chromosomal anomalies. Over 95% of the DFSP tumors have the chromosomal translocation t(17:22). The translocation fuses the collagen gene (COL1A1) with the platelet-derived growth factor gene. The tumor cells express the fusion gene. The resultant fusion protein is processed into a potent growth factor.[3]

Enzinger and Weiss developed a classification of soft tissue sarcomas on the basis of the histological appearance of the normal tissue. However, the World Health Organisation (WHO) classifies fibrous histiocytoma as a benign tumor and DFSP as an intermediate malignant tumor of fibrohistiocytic origin.[1] There are a number of histological variants of DFSP, which include fibrosarcomatous, myxoid, granular cell, atrophic, giant cell fibroblastoma and pigmented DFSP (Bednar tumor).[6] A fibrosarcomatous change in DFSP is characterized by the herringbone pattern of tumor cells, increased cellularity and increased mitoses (more than eight mitoses / 10 high power field). This change is associated with a more aggressive clinical course, indicating a need for intensive treatment. The myxoid variant of DFSP shows a less prominent storiform pattern and more conspicuous blood vessels.[6]

Histologically, DFSP should be differentiated from lesions, such as, benign and malignant fibrous histiocytoma, atypical fibrosarcoma, dermatofibrosarcoma, infantile myofibromatosis, nodular fasciitis, keloid, myxoid liposarcoma and neural tumors; all of which may have similar pathological findings.[1,3,5,7–9] Immunohistochemistry using CD34 is a useful marker for differentiation of DFSP and is also helpful in identifying tumor cells at surgical margins when treating recurrent DFSP. Other fibrohistiocytic lesions usually show negative CD34 expression.

Surgical excision is the treatment of choice. Long-term follow-up is needed, as one-third of the cases develop recurrence after five years.[1,2] When the margin of surrounding skin is less than 3 cm, the local recurrence rate is around 50%, which is almost always seen within two years. Regional and distant metastases from DFSP do occur, but are rare, probably less than 1%. According to other studies, the metastasis rate is under 5% in DFSP. Generally, distant metastasis occurs in the lung after recurrent local relapse and following regional lymph node involvement. The visceral organs and bones are rarely affected.[2,3,5,9] In our case, no sign of lymph node or distant organ involvement was seen.

The preferred therapy for DFSP is wide radical surgical excision. The technique should include resection of a 3 cm margin of skin beyond the borders of the tumor and should include the fascia and even the muscle tissue, if necessary.[2,5,8,9] Radiotherapy is of limited value in the treatment of DFSP. Moh's micrographic controlled surgery is the treatment of choice where extensive surgical excision of a DFSP tumor is not possible, that is, on the hand, foot, or areas of the face and head and neck region. Excision is extended till the frozen sections are negative for neoplastic cells.[9,10]

Late presentation, aggressive local invasion, regional lymph node involvement and distant metastases herald poor prognosis in addition to some histological features such as, increased mitotic figures, increased cellularity, DNA aneuploidy, TP53 gene expression and fibrosarcomatous change.[10]

The tumor presents a diagnostic challenge because of its rarity and its histological variants. Despite locally aggressive behaviour, this tumor infrequently metastasizes, and hence, it should be clearly distinguished from the conventional sarcomas. The correct diagnosis and treatment of choice offer a significant improvement in the cure rate, cosmetic advantage and patient's suffering.
